# Place of Death for People with Schizophrenia and Bipolar Disorder in New Zealand: A National Retrospective Cohort Study

**DOI:** 10.1177/08258597251339868

**Published:** 2025-05-13

**Authors:** Ruth Cunningham, Gawen Carr, Susanna Every-Palmer, Debbie Peterson, Tracy Haitana, Helen Butler, Anthony J O’Brien, Salina Iupati

**Affiliations:** 1 572298 Department of Public Health, University of Otago Wellington, New Zealand; 2 Mental Health, Addiction and Intellectual Disability Service, Te Whatu Ora, New Zealand; 3 626383 Department of Psychological Medicine, University of Otago Wellington, New Zealand; 4 572298 Department of Public Health, University of Otago Wellington, New Zealand; 5 Māori/Indigenous Health Institute (MIHI), 2494 University of Otago Christchurch, New Zealand; 6 School of Nursing, University of Auckland, Auckland, New Zealand; 7 3717 University of Waikato, Hamilton, New Zealand; 8 Palliative Medicine Consultant, 93869 Te Omanga Hospice, Wellington, New Zealand

**Keywords:** mental health, end of life, place of death, hospice, palliative care, schizophrenia, bipolar affective disorder, cohort study

## Abstract

**Objectives:** Inequities in access to physical health care for those with mental health conditions and substance use disorders are well recognised, and evidence of unequal access to palliative care is emerging. This study uses complete national data to examine the place of death for those with specific mental health conditions in Aotearoa New Zealand. **Methods:** Mortality data between 2013 and 2018 was linked to secondary mental health service usage. Place and cause of death were compared between those with diagnoses of bipolar affective disorder or schizophrenia and those without, stratified by ethnicity. **Results:** A cohort of 498,293 individuals was identified. People diagnosed with bipolar disorder and schizophrenia had different patterns of cause and place of death from other New Zealanders. This group was less likely to die in hospices, even after adjustment for differences in cause of death and age (adjusted OR 0.59, CI 0.52-0.68). Patterns of place of death differed by ethnicity. **Conclusions:** Inequities in healthcare provision for those diagnosed with psychotic disorders continue at the end of life, with reduced access to hospice facilities. Further research is needed to understand the quality of healthcare provision and wishes of those with mental health conditions in end-of-life care.

## Introduction

A good quality of life includes having access to effective and respectful healthcare throughout, including at life's end. In general, people living with mental health conditions such as bipolar disorder and schizophrenia have more medical comorbidities, receive lower-quality medical care, and die younger than the general population.^1^ They often experience unequal access to and quality of health care, which extends to end-of-life care. This may include reduced access to palliative care services,^2,3^ a lower likelihood of provision of analgesia prior to death,^4^ more fragmented care,^5^ and a lower level of advanced care planning.^6^

Common priorities for end-of-life health care include having one's preferences and autonomy respected, effective pain control, and support for emotional wellbeing.^7^ The few studies examining the wishes of people with serious mental illness have found similar end-of-life preferences to those without such illnesses,^8^ with both groups desiring access to palliative services. Palliative care is associated with clinically significant improvements in quality of life, improvements in advance care planning, higher patient and caregiver satisfaction, and lower health care utilisation.^9^ Palliative care should be accessible at home as well as in hospitals and in hospices, although complex care may not be practicable at home. However inpatient hospices and acute hospitals often are not designed to care for people with complex mental health needs, and mental health clinicians and mental health facilities may not be well equipped to provide end-of-life care in the community or in residential settings, creating barriers to palliative care for people with mental health conditions.^10^

Place of death is one aspect of end-of-life health care that may indicate access to palliative care. Inpatient hospices and acute hospitals are most likely to be settings with good access to palliative care, although palliative care is also provided in community and residential settings. Where place of death for those with serious mental illness has been investigated internationally, deaths in residential care settings have been found to be more common, but there is limited information about access to hospice care.^8^

In New Zealand, there are known inequities in mental health, mortality, and health care provision for Indigenous peoples (Māori) that could affect palliative care for Māori with mental health conditions. In general, Māori have higher rates of many mental health conditions than non-Māori, including psychosis and schizophrenia, but lower rates of treatment.^11^ For example, although mortality from natural causes is higher in Māori with severe mental illness than non-Māori, hospitalisation rates are not, signifying under-treatment of physical conditions.^12^ The effects of colonisation, discrimination, and differential access to the key social determinants of health underpin these inequities in mental health outcomes and also affects access to palliative care.^13^

This study aimed to investigate the location of end-of-life care among people with severe mental illness diagnoses using national routine data, in order to examine access to inpatient hospice care as an indicator of access to palliative care. The study compared the place of death between people with diagnoses of bipolar disorder or schizophrenia (BPSC) and those who had not used secondary mental health and addiction (MHA) services, using national death registration data, including the odds of inpatient hospice death, after adjusting for age, gender and cause of death. We also separately examined the relationship between place of death and a history of a diagnosis of bipolar disorder and schizophrenia separately among Māori and non-Māori in order to understand differences in the relationship between ethnic groups.

## Methods

### Data Source and Cohort Selection

The National Mortality Collection dataset, which holds data on location and cause of death, was used to identify all deaths in New Zealand between 2013 and 2018. The Programme of Integration for Mental Health Data (PRIMHD) dataset, which records service use and diagnoses for people accessing specialist MHA services, was used to identify all people using these services between January 2009 and December 2018. The Mental Health Information Collection dataset, the predecessor to PRIMHD, was used to identify diagnoses and treatment from 2001 to 2008. Death records were linked to MHA service use records using an encrypted unique identifier to identify all people who had three or more face-to-face contacts with services in the five years prior to death (people with evidence of active mental health or substance use conditions). People who were 10 years old or younger when they died were excluded.

### Variables

Demographic information (age at death, gender, and ethnicity) recorded on the National Mortality Collection was used. Ethnic identity as recorded in the Mortality collection was categorised into Māori (Indigenous) and a residual non-Māori (non-Indigenous) category which predominantly includes people of European ethnicity. Further break down of ethnic groups was limited by statistical power.

Mental health diagnoses were derived from specialist MHA service use records. Among the group in contact with specialist MHA services, people who had recorded diagnoses of schizophrenia or other non-organic psychoses or bipolar disorder (ICD10 codes: F31, F20-F25, F28, F29) were identified. This group was the main focus for the analyses presented in this paper. We chose to use the diagnoses of bipolar disorder and schizophrenia as our variables to define our population of interest. These diagnoses best resemble definitions of ‘serious mental illness’ used in the wider literature within the data we have available.^14^

Cause of death information was retrieved from the Mortality collection. Cause of death was categorised into Cancer; other chronic disease (cardiovascular, metabolic, neurological, respiratory causes); external causes (accidental and traumatic deaths, suicide and homicide); and other (causes of death not categorised as above, including infectious causes).

Place of death as recorded at death registration was retrieved from the Mortality collection and categorised into inpatient hospice, inpatient hospital, residential care, private residence, and ‘other’.

### Analyses

Analysis was completed using Stata s17. Descriptive analysis of causes of death and places of death was undertaken, for people with recorded diagnoses of BPSC compared with those who had not accessed specialist MHA services. The study was approved by the University of Otago Human Ethics Committee ref: HD20/080. We also consulted with the University's Ngāi Tahu Research Consultation Committee, which provides advice about conducting research to Māori, and worked in partnership with the Māori/Indigenous Health Innovation (MIHI) department (including author TH).

Multivariable logistic regression was used to compare the odds of death in a hospice setting between those with a diagnosis of BPSC and those without. Because the outcome of death in a hospice setting is rare (7%), the odds ratios provide a reasonable estimate of the relative risks of the outcome. Regressions were adjusted for confounding by age of death, gender, ethnicity and cause of death. Because analysis showed a modification of the relationship between place of death and bipolar or schizophrenia diagnosis by ethnicity, final results are stratified by ethnicity.

Sensitivity analyses were conducted against broader definitions of serious mental illness. The first comparison group included not only those with diagnoses of bipolar and schizophrenia but anyone with a period of treatment in hospital or treated under compulsory mental health legislation at any point since 2001. The second comparison group included all people, regardless of diagnosis, who had three or more contacts with specialist MHA services in the 5 years prior to death. Further sensitivity analyses used restricted datasets, firstly restricting to deaths from chronic conditions only, as only deaths that are expected (such as from chronic conditions) can be referred to palliative care. A final sensitivity analysis limited the cohort to those who died under the age of 65, as MHA services provided to those over 65 are not consistently and completely recorded in the PRIMHD dataset, with regional variation in reporting. Therefore not everyone aged over 65 with active contact with MHA services will be identified in PRIMHD.^15^

## Results

The total sample comprised 498,293 people who had died between 2013 and 2018. Of this number, 7898 people had recorded diagnoses of BPSC group, and 449,255 who did not, and who had not accessed secondary MHA services (control group). The age distribution of identified deaths was younger among Māori compared to non-Māori, and among those with BPSC compared to the control population.

The patterns of *cause of death* differed according to mental health status and ethnicity (Table 1). Overall people with BPSC had fewer deaths recorded from cancer (18% vs 30%) or other chronic diseases (49% vs 54%), and more deaths recorded from external causes (21% vs 7%).

**Table 1.** Demographic Characteristics and Place and Cause of Death for People Who Died in Aotearoa New Zealand Between 2013 and 2018, by Ethnicity and History of Mental Health Conditions.

	Māori		Non-Māori		Total
	BPSC^a^	Control	BPSC^a^	Control	
	*n* (%)	*n* (%)	*n* (%)	*n* (%)	*n* (%)
Total sample	1438 (100)	47,600 (100)	6460 (100)	442,795 (100)	498,293 (100)
Age at death					
<55 years	810 (56)	13,135 (28)	1614 (25)	36,509 (8)	52,068 (10)
55–74 years	505 (35)	21,501 (45)	2370 (37)	110,060 (25)	134,436 (27)
≥75 years	123 (9)	12,964 (27)	2476 (38)	296,226 (67)	311,789 (63)
Gender					
Female	661 (49)	22,119 (46)	3299 (51)	223,095 (50)	249,174 (50)
Male	777 (51)	25,481 (54)	3161 (49)	219,700 (50)	249,119 (50)
Prioritised ethnicity^b^					
Māori	1438	47,600	-	-	49,038 (10)
Pacific	-	-	368 (6)	18,074 (4)	18,442 (4)
Pākehā/NZ European	-	-	5893 (91)	410,359 (93)	416,252 (84)
Other	-	-	199 (3)	14,362 (3)	14,561 (2)
Cause of death					
Cancer^c^	265 (18)	15,005 (32)	1173 (18)	132,482 (30)	148,925 (30)
Other chronic disease^d^	627 (44)	23,717 (50)	3180 (49)	241,261 (54)	268,785 (54)
External causes^e^	383 (27)	5525 (12)	1349 (21)	3,2884 (7)	40,141 (8)
Other^f^	163 (11)	3353 (7)	758 (12)	3,6168 (8)	49,442 (8)

a BPSC those with recorded diagnoses of bipolar disorder or schizophrenia. BP is bipolar affective disorder, SC is schizophrenia.

b Ethnicity is reported in the death certificate data and is prioritised in the following order: Māori, Pacific, Asian, Other, NZ European.

c ICD10 codes: C00-D49.

d ^‘^Other chronic disease’ does not include cancer, and comprises mainly cardiovascular, metabolic, neurological, respiratory causes. ICD10 codes D50-D89, E00-E89, G00-G99, I00-I99, J00-J99, K00-K93.

e ^‘^External causes’ include accidental and traumatic deaths, suicide and homicide. ICD10 codes S00-T88, V01-Y89.

f ‘Other’ causes of death include infectious causes. All remaining ICD10 codes.

**Figure 1.**
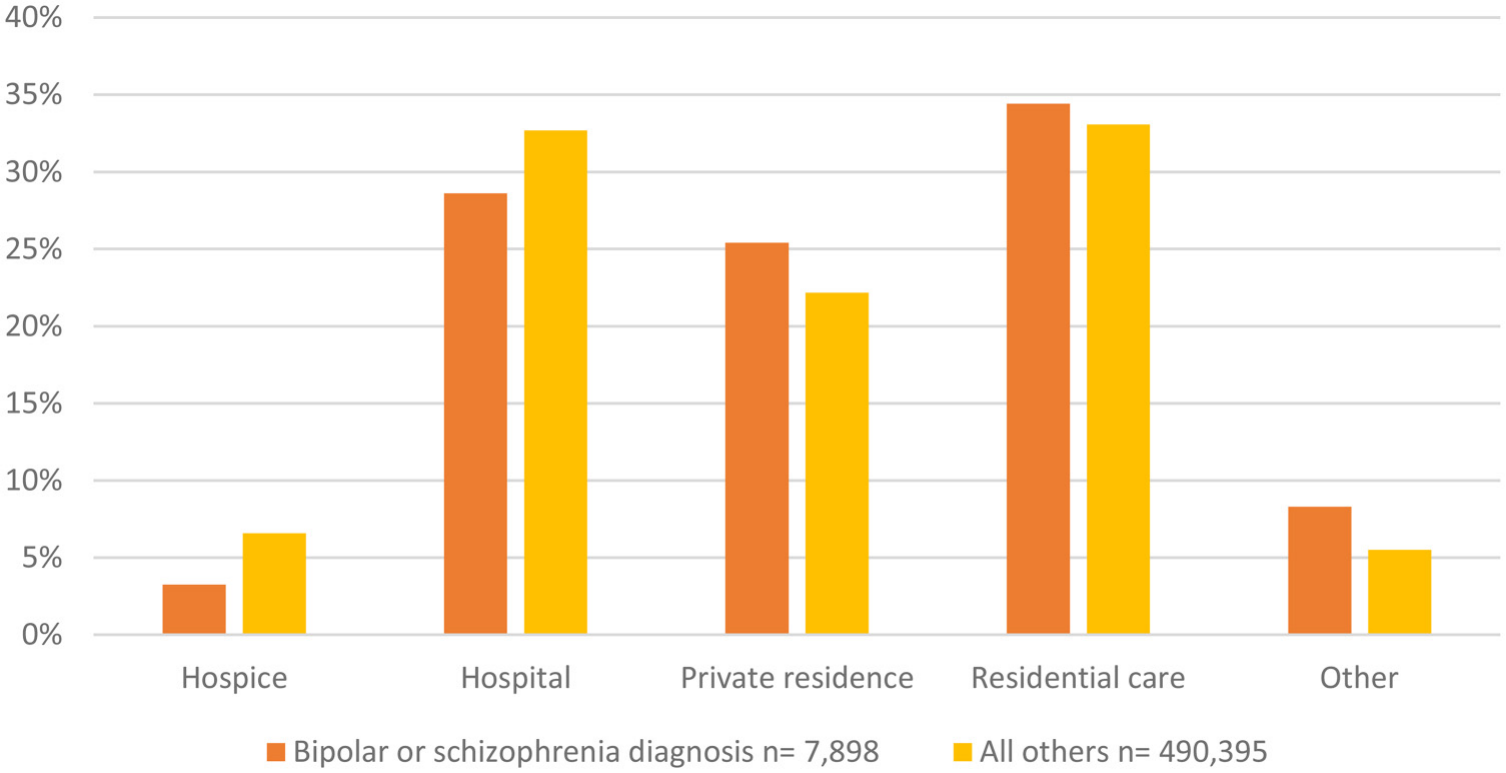
Recorded place of death by mental health diagnosis.

There were marked differences in patterns of *place of death* according to mental health status (Figure 1). People with BPSC diagnoses were less likely to die in a hospice (3% vs 7%), less likely to die in hospital (28% vs 32%), more likely to die in a private residence (22% vs 20%) and more likely to die in residential care (39% vs 35%) compared to people without a history of these mental health diagnoses.

**Figure 2.**
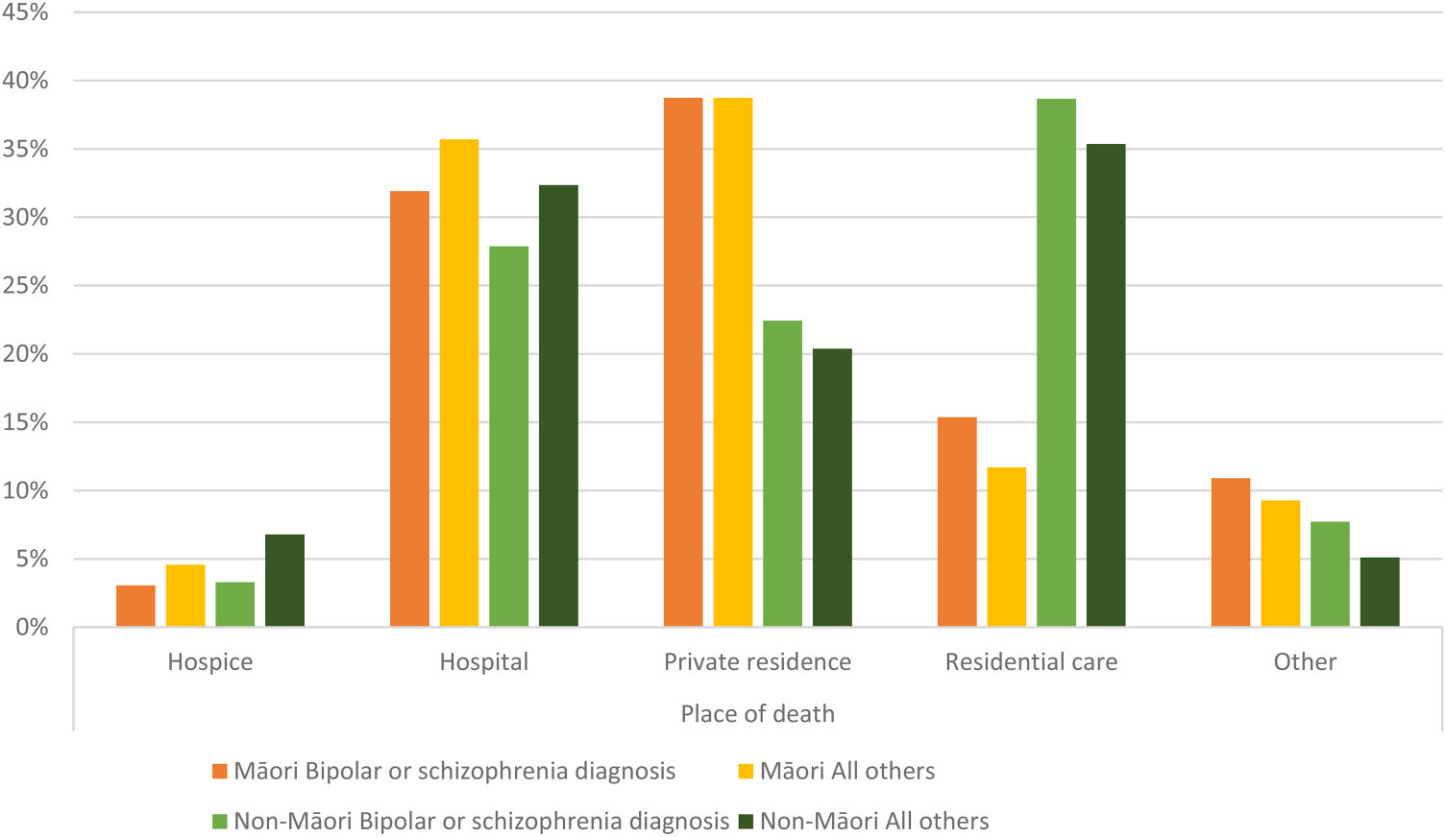
Patterns of place of death by ethnicity and mental health diagnosis.

When further examined by ethnicity (Figure 2), marked differences between Māori and non-Māori were also apparent. Regardless of mental health diagnosis, Māori had markedly different patterns of place of death compared to non-Māori. Specifically, dying at home was much more common among Māori while non-Māori were much more likely to die in residential care settings. Differences between Māori with BPSC diagnoses and those without followed similar patterns to non-Māori, with a lower proportion of those with BPSC dying in hospice or hospital settings.

Table 2 shows that the odds of someone with a diagnosis of BPSC dying in hospice was 0.59 (95% CI: 0.52-0.68) compared to people without these diagnoses, after adjusting for confounding by demographic factors and cause of death. The odds of Māori dying in hospice care was 0.46 (95% CI: 0.52-0.68) compared to non-Māori, after adjustment for age, gender and cause of death. There was a significant interaction effect (*p* = 0.016)), suggesting an effect modification by ethnicity in the relationship between mental health diagnoses and odds of dying in hospice (that is, there are different relationships between mental health diagnoses and place of death for Māori and non-Māori). We, therefore, modelled the odds of dying in hospice separately for Māori and non-Māori. Māori with diagnoses of BPSC had similar odds of dying in hospice care compared to Māori without these diagnoses (OR = 0.91, 95% CI: 0.66-1.24), while a stronger difference by mental health diagnosis was found when non-Māori were separately examined (OR 0.56, 95% CI 0.48-064).

**Table 2.** Adjusted odds of dying in hospice by bipolar or schizophrenia diagnosis and by ethnicity.

	Crude % dying in hospice		Odds ratio	95% Confidence interval
	BPSC	Non-BPSC		
BPSC versus non-BPSC^a^	3.3%	6.6%	0.59	0.52–0.68
	Māori	Non-Māori		
Māori versus non-Māori^b^	4.5%	6.7%	0.46	0.44–0.48
	Māori with BPSC	Māori no BPSC		
Māori with BPSC versus Māori without BPSC^c^	3.1%	4.6%	0.91	0.66–1.24
	Non-Māori BPSC	Non-Māori no BPSC		
Non-Māori with BPSC versus Non-Māori without BPSC^a^	3.3%	6.8%	0.56	0.48–0.64

a Adjusted for age at death, gender, ethnicity and cause of death.

b Adjusted for age at death, gender, cause of death and mental health diagnoses.

c Estimate of the OR for BPSC versus non-BPSC stratified by ethnicity, adjusted for age at death, gender and cause of death.

Lower access to hospice inpatient units persisted among non-Māori with BPSC when the analysis was restricted to deaths from medical (chronic disease) causes (Supplemental Table 1). We also tested whether the relationship varied by diagnoses of mental health condition and found that having a serious mental illness by all definitions tested meant a lower likelihood of a hospice death for non-Māori (Supplemental Table 2). Restricting our analysis to deaths occurring before the age of 65 (Supplemental Table 3) did not change the pattern of the results.

## Discussion

This study shows that New Zealanders with diagnoses of BPSC die in different places to other New Zealanders, indicating that inequities seen in physical health care for people with mental health and substance use conditions persist to the end of life.

We found that people with BPSC were less likely to die in hospices than the rest of the population. Physical health disparities and premature mortality are observed globally among those with mental health and substance use conditions,^1^ but there is sparse evidence and less consistency in patterns of end-of-life health care for people with these diagnoses.^8^ A recent systematic review of studies examining end-of-life care and place of death in adults with serious mental illness identified six studies examining receipt of palliative care. Four of these found a lower likelihood of palliative care receipt than the general population, but no studies examined the likelihood of hospice as a place of death.^8^ A previous New Zealand regional study, included in this review, found people with serious mental illness were 3.5 times less likely to receive specialist palliative care,^2^ in line with our results.

In a study of end-of-life preferences of elderly New Zealanders, the top priority for both Māori and Non-Māori participants was not being a burden to their family.^16^ Unlike international studies, a home death was not a high priority for either group. In another study,^17^ families regarded good end-of-life care as profoundly relationship-driven and upholding the dying person's mana (dignity, authority). More detailed information than simply place of death is required to understand whether end-of-life care for people with serious mental illness is in line with their preferences and priorities.

The discrepancies in hospice deaths may both be a consequence and a perpetuating cause of the inequitable health care that people with bipolar disorder and schizophrenia experience throughout their lives. Previous research shows people do not differ appreciably in their preferences for end-of-life care depending on whether they have a serious mental illness or not.^18,19^ Although there are a number of possible causes for the findings beyond different preferences to care, it is likely that the finding that fewer people with bipolar disorder and schizophrenia received hospice care is at least in part a result of barriers to care and structural discrimination. Unequal health care provision is known to contribute to poor health outcomes for people with bipolar disorder and schizophrenia^1^ which includes poorer access to palliative care services.^20^ Structural discrimination contributes to poorer access to care through stigma, a lack of recognition and management of physical health conditions by health providers, unfamiliarity in how to communicate and implement advance care planning or include non-traditional wider support networks and limited understanding and collaboration with palliative care from mental health providers.^21,22^ People with bipolar disorder and schizophrenia may not trust that physical and end-of-life health care needs can be met due to previous adverse experiences of health services.^23^

The finding of a different pattern for Māori, where death in a hospice setting was uncommon regardless of mental health history, likely reflects other factors which impact on Indigenous people's access to appropriate end-of-life care. For example, physical access to hospice services in New Zealand is limited for rural populations including Māori. Discrimination also acts as a barrier for Māori, whose experience of racism within the health system can undermine trust. Western palliative care services may not be seen as culturally safe or appropriate or aligned with traditional practices of whānau/family caregiving. Moreover, there is increasing evidence that Māori do not receive services at the end of life commensurate with need, and that families often feel unsupported during caring and bereavement, indicating a gap in service provision for Māori.^13,20^

## Strengths and Limitations

The strengths of this paper include the large sample, comprised of a complete national dataset of linked mortality and specialist MHA service use data. We were able to adjust for key confounding factors, and to conduct sensitivity analyses using different definitions of mental illness, restricted causes of death, and restricted age of death to exclude these factors as being causes for the relationships observed.

However, such routine data use has limitations. Coding on place of death was derived from death certificates, which sometimes lack detail when completed in the community, making it difficult to differentiate between settings such as private units in residential villages and high-dependency nursing homes. It was also not possible to identify cases where death occurred in home or hospital settings under the care of a hospice or hospital palliative care team. As most specialist palliative care occurs outside of inpatient hospice settings, we were not able to see the full picture of access to specialist palliative care in this study. Much of palliative care occurs in primary care which could not be captured in this study. Data from secondary MHA services was used to identify serious mental illness, and so only those who had accessed secondary MHA services in the five years prior to death were included in the group with schizophrenia or bipolar disorder. Those who were managed in primary care, or who had not recently had contact with services would not be identified. The results are therefore not generalisable to those with serious mental illness not currently or recently in contact with specialist services. Moreover, within the PRIMHD dataset, data on both diagnoses and secondary MHA service contact in those aged over 65 are incomplete, both of which could result in misclassification bias by underestimating the size of the group with bipolar disorder and schizophrenia. However our sensitivity analyses, including people with secondary MHA service contact without relying on diagnosis data, and excluding all those over 65 at the time of death, both demonstrated the same relationship between serious mental illness and likelihood of hospice as place of death.

## Implications

Addressing health inequities impacting people with serious mental illness at the end of their life has been reviewed and reported elsewhere.^10,24^ Key priorities involve close partnerships between mental health, addiction and end-of-life care systems, supported decision making including advance planning for end-of-life care, recognising and addressing social needs, and ensuring ongoing MHA care.^10,24^ Our data indicate that such partnerships need to be developed in Aotearoa New Zealand.

Services for people with mental health and substance use conditions at the end-of-life require a team approach including advocacy, and proactive physical health care to tackle the problems of delayed or suboptimal treatment.^10^ Discrimination and diagnostic overshadowing are serious issues for New Zealanders with mental health and substance use conditions and can lead to avoidance of general health treatment.^25,26^ It is likely this extends to end-of-life care. In an Australian study, 89% of hospital doctors endorsed at least one stigmatising or discriminatory attitude toward people with mental illness with respect to end-of-life care.^27^ Education, support and supervision for staff is therefore required, together with monitoring equity of service provision for people with mental health and substance use conditions across the entire health care continuum.

The findings of our study starkly highlight the differences in where Māori die compared to non-Māori New Zealanders. For Māori both with and without diagnoses of BPSC, hospice was an uncommon place of death and the majority of people died in a private residence. While our other work has found that physical health care inequities experienced by people with mental health and substance use conditions are amplified among Māori with these conditions,^12,28^ in the case of place of death, Māori with schizophrenia and bipolar disorder did not differ markedly from Māori without these conditions. Other research has suggested that palliative care services are not well equipped to meet the needs of Māori,^29–31^ and a Māori palliative care framework for hospices has recently been developed.^32^ The applicability of this framework, and the particular needs of Māori with mental health and substance use conditions for end-of-life care, is an important target for further research.

## Conclusion

For all New Zealanders, end-of-life care, and place of death should reflect individual choice, autonomy and preferences. Our results suggest that for people with diagnoses of BPSC, inequities exist in access to end-of-life care that is likely a consequence of inequities in health care provision throughout their lifespan. Palliative care is a resource that should be available equitably, irrespective of disability or illness.

## Supplemental Material

sj-docx-1-pal-10.1177_08258597251339868 - Supplemental material for Place of Death for People with Schizophrenia and Bipolar Disorder in New Zealand: A National Retrospective Cohort Study Supplemental material, sj-docx-1-pal-10.1177_08258597251339868 for Place of Death for People with Schizophrenia and Bipolar Disorder in New Zealand: A National Retrospective Cohort Study by Ruth Cunningham, Gawen Carr, Susanna Every-Palmer, Debbie Peterson, Tracy Haitana, Helen Butler, Anthony J O’Brien and Salina Iupati in Journal of Palliative Care
