# Supplementary material for: Place of Death for People with Schizophrenia and Bipolar Disorder in New Zealand: A National Retrospective Cohort Study
Source: J Palliat Care. 2025 May 13;41(2):151–8. doi: 10.1177/08258597251339868 (PMC13013657; doi:10.1177/08258597251339868)
Supplement: sj-docx-1-pal-10.1177_08258597251339868 - Supplemental material for Place of Death for People with Schizophrenia and Bipolar Disorder in New Zealand: A National Retrospective Cohort Study [file sj-docx-1-pal-10.1177_08258597251339868.docx]

## Supplementary tables

**Table 1: Sensitivity analysis 2: Odds of hospice use in those with BPSC, compared to those with no MHA service use, for death due to cancer and chronic disease only**

|  | Māori | | Non-Māori | |
| --- | --- | --- | --- | --- |
|  | Odds ratio | Confidence interval | Odds ratio | Confidence interval |
| All Causes of death | 0.91 | (0.66 - 1.24) | 0.56 | (0.48 - 0.64) |
| Death from cancer + chronic disease | 1 | (0.73 - 1.37) | 0.57 | (0.49 - 0.66) |

**Table 2: Sensitivity analysis 1: Odds of hospice use in those with different definitions of severe mental illness, compared to those with no MHA service use**

|  | Māori | | Non-Māori | |
| --- | --- | --- | --- | --- |
|  | Odds Ratio | Confidence interval | Odds Ratio | Confidence interval |
| Bipolar or schizophrenia diagnosis (BPSC) | 0.91 | (0.66 - 1.24) | 0.55 | (0.48 - 0.64) |
| BPSC + inpatient + care under Mental Health Act | 1.08 | (0.84 - 1.39) | 0.60 | (0.54 - 0.67) |
| All users of specialist mental health and addiction services | 1.17 | (1.00 - 1.38) | 0.69 | (0.65 - 0.74) |

**Table 3: Sensitivity analysis 3: Odds of hospice use those with BPSC compared to those with no MHA service use, for people under 65 years of age**

|  | Māori | | Non-Māori | |
| --- | --- | --- | --- | --- |
|  | Odds ratio | Confidence interval | Odds ratio | Confidence Interval |
| All deaths | 0.91 | (0.66 - 1.24) | 0.56 | (0.48 - 0.64) |
| Aged under 65 years | 0.93 | (0.65 - 1.32) | 0.65 | (0.54 - 0.79) |
